# Determination of Multi-Class Mycotoxins in Tartary Buckwheat by Ultra-Fast Liquid Chromatography Coupled with Triple Quadrupole Mass Spectrometry

**DOI:** 10.3390/toxins10010028

**Published:** 2018-01-04

**Authors:** Guixing Ren, Yichen Hu, Jinming Zhang, Liang Zou, Gang Zhao

**Affiliations:** 1 Key Laboratory of Coarse Cereal Processing, Ministry of Agriculture, Chengdu University, Chengdu 610106, China; renguixing@cdu.edu.cn (G.R.); zouliang@cdu.edu.cn (L.Z.); zhaogang@cdu.edu.cn (G.Z.); 2 Institute of Crop Sciences, Chinese Academy of Agricultural Sciences, Beijing 100081, China; 3 College of Pharmacy, Chengdu University of Traditional Chinese Medicine, Chengdu 611137, China; zhangjinming1987@126.com

**Keywords:** UFLC-QTrap-MS/MS, mycotoxins, Tartary buckwheat, matrix effect, aflatoxin, This study established a simple, rapid and cost-effective analytical method for multi-class mycotoxins determination in Tartary buckwheat based on dilute-and-shoot pretreatment coupled with ultra fast liquid chromatography-tandem mass spectrometry (DAS-UFLC-MS/MS). It would provide a useful analysis approach for mycotoxins quantification in foodstuff.

## Abstract

Considering crops are susceptible to toxicogenic fungi during plantation, pre-processing and storage, an ultra-fast liquid chromatography coupled with triple quadrupole mass spectrometry (UFLC-QTrap-MS/MS) method was developed and validated for simultaneous determination of the 12 most frequent mycotoxins, including aflatoxin B_1_, B_2_, G_1_, G_2_, HT-2, T-2 toxin, ochratoxin A, fumonisin B_1_, B_2_, zearalanone, zearalenone, and deoxynivalenol, in 14 batches of Tartary buckwheat cultivar, collected from different origins in Sichuan Province, China. Differing from those complicated approaches, a simple and cost-efficient pretreatment method based on dilute-and-shoot was employed. Based on optimized chromatographic and mass spectrometry conditions, these 12 mycotoxins could be analyzed with high correlation coefficients (all over 0.995), high precision (RSD 0.47–9.26%), stability (RSD 0.72–11.36%), and recovery (79.52% to 108.92%, RSD 4.35–14.27%). Furthermore, this analysis method exhibited good determination performance with little disturbance of the matrix effect. Finally, this proposed method was applied for 14 batches of Tartary buckwheat seeds, in which aflatoxin B_1_ (AFB_1_) was detected in one moldy cultivar, Meigu No. 2, with its concentration exceeding the maximum residue limits set by EU regulations. The method thus established, which has significant advantages, could provide a preferred determination approach candidate for measurement of multiple mycotoxins measurement in Tartary buckwheat, even other kinds of foodstuffs.

## 1. Introduction

Tartary buckwheat (*Fagopyrum tataricum* Gaertn.), also known as bitter buckwheat, is an excellent edible and medicinal crop mainly grown in the mountainous regions of Southwest China, northern India, Bhutan, Nepal and Slovenia [1,2]. The nutritional value of Tartary buckwheat seeds as a kind of corn has been well acknowledged, as it is a rich source of carbohydrates, protein, vitamins, dietary fibre, and minerals. Nevertheless, compared with other common buckwheat species, e.g., sweet buckwheat (*F. esculentum* Moench) and golden buckwheat (*F. dibotrys* (D. Don)), the renewed and increasing interest in the utilization of Tartary buckwheat relies on various health benefits, such as anti-hypertension [3], anti-fatigue [4], cholesterol-lowering [5], and so on. These bioactivities can be attributed to the bioactive phytochemicals it contains, e.g., flavonoids [6,7]. A number of both epidemiological and basic studies have validated its essential health. For this reason, the production of Tartary buckwheat around the world is rising [8]. Especially in China, various Tartary buckwheat food products, including tea, vinegar, noodles and cakes, are becoming increasingly popular. 

However, like many other crops, buckwheat has recently been included in the list of cereals and derived products that may be susceptible to invasion by toxigenic fungi, such as Aspergillus flavus, Fusarium, and Penicillium, during planting or storage [8,9]. These toxigenic fungi are not only considered significantly harmful pathogens in terms of their influence on the quality, safety and efficacy of Tartary buckwheat, but they also result in mycotoxin contamination (EFSA, Supporting Publications 2013: EN-406) [10]. Multi-class mycotoxins, mainly comprising aflatoxins (AF), ochratoxins (OT), fumonisins, zearalenone (ZEA), and trichothecenes, have been found as secondary fungal metabolites in these fungus-contaminated food or crops [8,11]. These mycotoxins pose serious public health issues, which have been demonstrated to include severe inducement of malignant neoplasm, mutagenicity, teratogenicity, nephrotoxicity, hepatotoxicity and dermatitis [12]. Aflatoxin B_1_ (AFB_1_) and its metabolic precursor sterigmatocystin, in particular, have been identified as being Class I carcinogenic by the World Health Organization’s (WHO) International Agency for Research on Cancer (IARC) Monographs Program. Most importantly, due to their high liposolubility and thermo-stability, mycotoxins are not only a problem during cropping, but also during the storage, transport, processing, and cooking steps [13]; aflatoxins remain essentially unaltered during food processing, such that the residue of these aflatoxins in buckwheat could be present in the resulting processed foods [14]. Therefore, the development of reliable and sensitive methods for accurately determining multi-class mycotoxins in buckwheat is urgently required in order to ensure its safety and quality. 

In view of the trace amounts of mycotoxins in foodstuffs, methods for their quantitative analysis must be both sensitive and selective. Traditionally, mycotoxin determination has mainly been performed by chromatographic techniques, including HPLC-UV/Vis [15], HPLC-FLD [16] or liquid chromatography/mass spectrometry (LC/MS) [17], in combination with effective separation approaches to avoid matrix disturbance. Most pretreatment approaches involve liquid-liquid extraction [18], solid-phase extraction [19], liquid-phase microextration [20] or an immune-affinity column [21]. However, these methods typically suffer from some limitations, such as low sensitivity, poor repeatability, and increased cost and time. Moreover, the complex matrix and diversity of mycotoxin structures results in the determination of multi-class mycotoxins by these pretreatment and analysis methods being highly challenging. Fortunately, a novel pretreatment approach, named “dilute-and-shoot”, whereby crude sample extracts are diluted without further clean-up, has been employed for high-throughput analysis of multi-mycotoxins in crops or herbs, and could minimize the matrix effect. To date, there has been no report that quantitatively determines the multi-mycotoxin in Tartary buckwheat simultaneously using the “dilute-and-shoot” pretreatment method [22].

**Figure 1:**
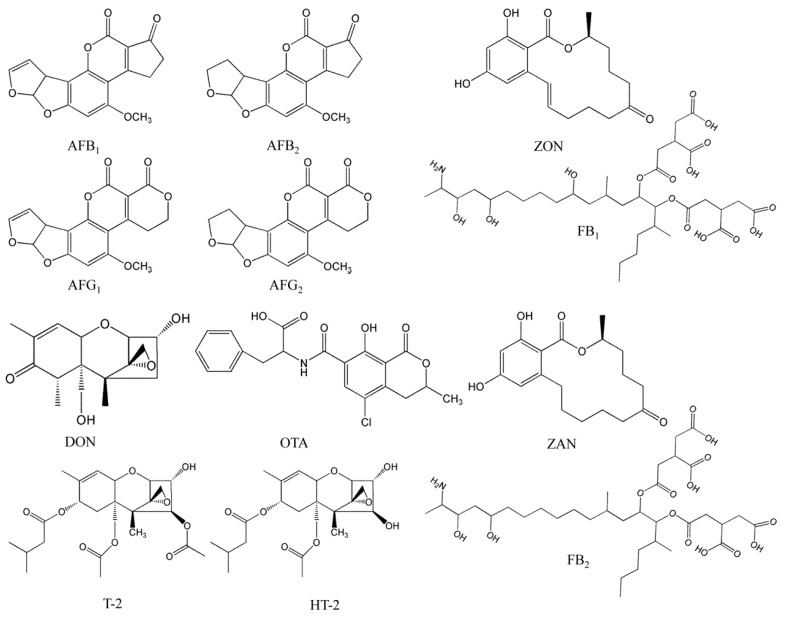
Chemical structure of the twelve analyzed mycotoxins.

The aim of this study was to establish a simple and rapid methodology based on LC-MS/MS for the simultaneous determination of 12 mycotoxins (Figure 1), including aflatoxin B_1_, B_2_, G_1_, G_2_ (AFB_1_, AFB_2_, AFG_1_ and AFG_2_), HT-2, T-2 toxin (HT-2, T-2), ochratoxin A (OTA), fumonisin B_1_, B_2_ (FB_1_, FB_2_), zearalanone (ZAN), zearalenone (ZON), deoxynivalenol (DON) in 14 batches of Tartary buckwheat. This approach, in combination with the UFLC-QTrap-MS/MS method, exhibits the advantages of being cost-effective, time-efficient and easy- to use. To the best of our knowledge, this is the first report on the evaluation and optimization of a reliable LC-MS/MS method for simultaneous detection and accurate determination of multi-class mycotoxins in Tartary buckwheat from different production origins.

## 2. Results and Discussion

### 2.1. Optimization of UFLC-QTrap-MS/MS Analysis Condition

Commonly, the diverse physico-chemical characters of different analytes greatly impede the development of methods for simultaneous quantitative analysis of multi-class mycotoxins. Initially, the elution conditions in the UFLC system were evaluated, whereby elution mobile phase composition and flow rate were optimized in view of the high sensitivity and satisfactory separation efficiencies of the 12 mycotoxins. Compared with conventional HPLC, UFLC has a relatively short run time and enhanced sensitivity and peak resolution, which has been applied to the determination of mycotoxins in herbs [23] and food [24]. According to the previous study [25], methanol and acetonitrile were the most frequently used organic phases in mycotoxin analysis. A variety of mobile phases, comprising methanol/acetonitrile as an organic phase and a water phase containing formic acid/ammonium formate/ammonium acetate, were investigated, including (a) water containing 10 mmol/L ammonium acetate, (b) water containing 0.1% formic acid, (c) water containing 0.2% aqueous ammonia, and (d) water containing 0.2% aqueous ammonia and 10 mmol/L ammonium acetate. The pre-experiment results indicated that acetonitrile was able to achieve better resolution and sensitivity for these 12 mycotoxins in comparison to methanol. Moreover, taking the high ion response during mass spectrum determination into consideration, 0.1% formic acid was chosen as the water phase. Moreover, the addition of 0.1% formic acid also benefits the separation of zearalenone and zearalenone, to avoid the overlap. Meanwhile, the acetonitrile-0.1% formic acid system was able to avoid the drastic ion signal fluctuation caused by the varying elution phase during gradient elution. Additionally, the flow rate of the mobile phase was also optimized at 350 μL·min^−1^, suggesting a good compatibility among sensitivity, resolution and analysis time.

Subsequently, the MS/MS parameters were optimized by infusing each mycotoxin into the mass spectrometer individually. Both positive and negative ionization modes were carried out, and the appropriate determination mode with the higher response was selected. As a result, among these mycotoxins, ZON, ZAN, and DON exhibited a higher response in the negative ionization mode [M − H]^−^. The rest of the mycotoxins showed a more satisfactory profile in [M + H]^+^ mode. Therefore, based on this, the optimum MRM transitions for each analyte were obtained by positive/negative polarity switching along the chromatographic run. The MS/MS parameters, including quantification and identification transition ion, declustering potential (DP), collision energy (CE), collision cell exit potential (CXP), and so on for the 12 target mycotoxins have been listed in Table 1.

### 2.2. Peak Identification

**Figure 2:**
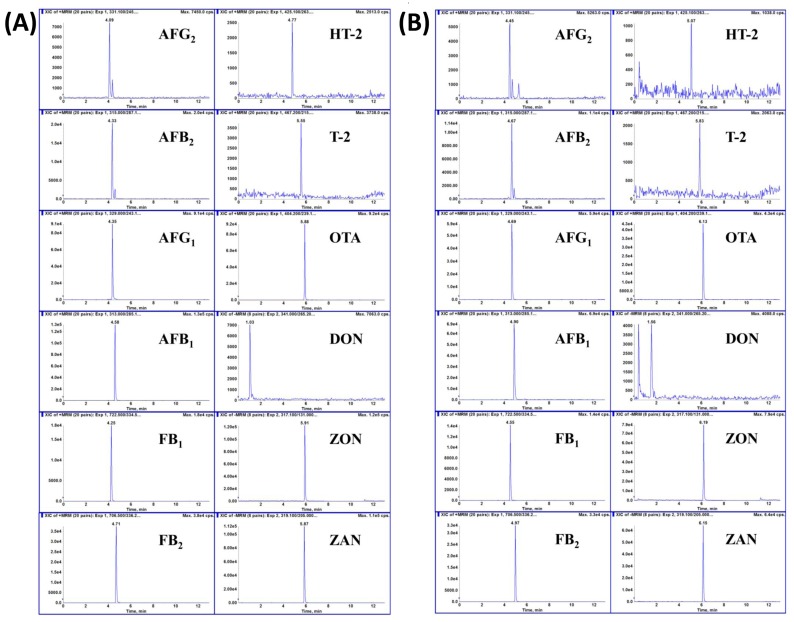
Typical UFLC-QTrap-MS/MS MRM chromatograms of (**A**) 12 mycotoxins in standard solutions (10 ng·mL^−1^ for AFB_1_ and AFG_1_, 2.5 ng·mL^−1^ for AFB_2_ and AFG_2_, 10 ng·mL^−1^ for OTA, 50 ng·mL^−1^ for ZON, ZAN, FB1, FB2, HT-2 and T-2, 100 ng·mL^−1^ for DON); (**B**) blank sample spiked with (10 μg·kg^−1^ for AFB_1_ and AFG_1_, 2.5 μg·kg^−1^ for AFB_2_ and AFG_2_, 10 μg·kg^−1^ for OTA, 50 μg·kg^−1^ for ZON, ZAN, FB1, FB2, HT-2 and T-2, 100 μg·kg^−1^ for DON).

In sample detection, retention time drift and interferences in the Tartary buckwheat matrix may increase the difficulty of identifying the analytes. Therefore, identification of the 12 mycotoxins was achieved by searching for peaks in the appropriate retention time windows (RTWs), defined as the retention time ± three standard deviations of the retention time for 10 blank samples (Cultivar Xiqiao No. 3) spiked at low level. In addition, the consistency of the ion ratios for two transitions (quantifier and qualifier transition ions) was also monitored and evaluated according to Commission Decision 2002/657/EC. The quantifier transition ion chromatograms of the 12 mycotoxins in standard solutions and in spiked blank sample solutions are shown in Figure 2.

### 2.3. Method Validation

Based on the above-optimized analysis conditions, the selectivity of the established method was firstly evaluated. The blank Tartary buckwheat methanol extraction solution, the 12-mycotoxin standard solution, and the mixture sample were analyzed. Consequently, no interfering peaks were found at the same retention time in blank Tartary buckwheat sample, suggesting the good selectivity of the established method for the determination of the 12 mycotoxins. 

The significance of the matrix effect at an individual concentration is dependent on the percentage differences between the average response of the mycotoxin in matrix and in solvent. The average matrix effect is regarded to be acceptable at ranges below 20%; otherwise, it is generally considered to exhibit an obvious effect on the quantitative analytical results. From the UFLC-Qtrap-MS/MS MRM chromatograms of the standard solution of mycotoxins and spiked blank Tartary buckwheat sample in Figure 2, and the value of signal suppression/enhancement (SSE) for selected mycotoxins in Table 2, it can be concluded that no obvious matrix effect was observed for the MS/MS response of mycotoxins.

The calibration curves of all mycotoxins spiked with blank matrix were performed and constructed. As shown in Table 2, good linearity was obtained, with all correlation coefficients having a value of (r) > 0.995 in the test ranges. Additionally, non-contaminated Tartary buckwheat was sampled and spiked with different concentrations of mycotoxins to measure the sensitivity of the method. LOQs (S/N = 3) of all mycotoxins were 0.1 ng·mL^−1^ for AFB_1_, 0.25 ng·mL^−1^ for AFB_2_ and OTA, 0.5 ng·mL^−1^ for AFG_2_, 1 ng·mL^−1^ for AFG_1_, FB_2_ and ZON, 5 ng·mL^−1^ for FB_1_, 10 ng·mL^−1^ for ZAN, and 50 ng·mL^−1^ for HT-2, T-2 and DON. LODs (S/N = 10) of all mycotoxins were 0.05 ng·mL^−1^ for AFB_1_, 0.1 ng·mL^−1^ for OTA, 0.125 ng·mL^−1^ for AFB_2_, 0.25 ng·mL^−1^ for AFG_2_, 0.5 ng·mL^−1^ for AFG_1_, FB_2_ and ZON, 2.5 ng·mL^−1^ for FB_1_, 20 ng·mL^−1^ for HT-2 and T-2, and 25 ng·mL^−1^ for DON. In view of the fact that all of these values are lower than the maximum residue levels established by the EU, this result suggests a good sensitivity for the proposed method. 

Meanwhile, precision, stability and recovery of this method were also investigated by analyzing the variation of a blank Tartary buckwheat sample spiked with all 12 mycotoxin standards in different situations. The intra-day variation, i.e., the sample extract was successively analyzed within a single day under the same conditions, was employed to characterize the precision of the instrument. Results indicated RSDs at different analysis times ranged from 0.47% to 9.26%, suggesting a high level of precision. Additionally, the mixed samples were injected into the analysis system at 0, 2, 4, 8, 16, 24, 48, 72 h to investigate the inter-day precision of determination of these mycotoxins in the Tartary buckwheat sample. As a result, the RSDs of these analyses ranged from 0.72% to 11.36%, indicating satisfactory inter-day precision. The stability evaluation results at 72 h were lower than 10%, suggesting samples remained stable for 3 days.

A recovery experiment was performed to evaluate the accuracy of the method by standard addition. The results of recovery (high, medium and low levels in triplicate) of mycotoxins are summarized in Table 3. It can be seen that the average recovery ranged from 79.52% to 108.92% for all mycotoxins (RSDs of these analytes ranged from 4.35% to 14.27%), which is in good agreement with Commission Regulation (EC) No 401/2006 for the performance criteria for quantitative methods of mycotoxin analysis, according to which all recovery values should be within 70–110%, with associated RSDs of less than 15%.

### 2.4. Determination of Tartary Buckwheat Samples

**Figure 3:**
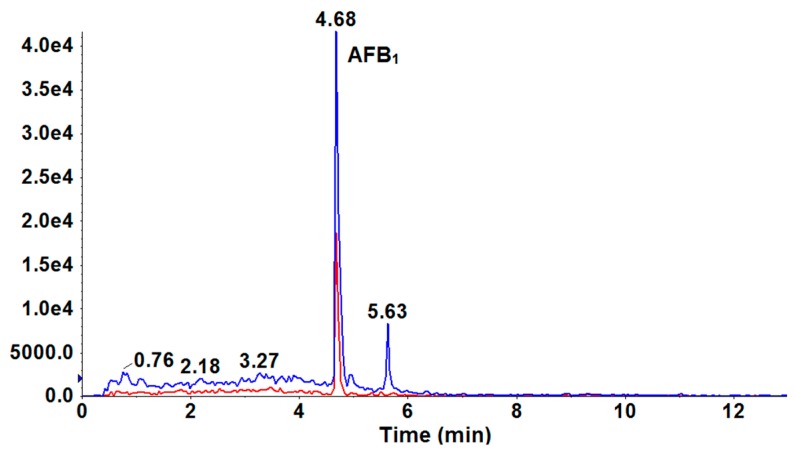
UFLC-MS/MS MRM chromatogram for AFB_1_ residue determination in contaminated Tartary buckwheat sample.

Finally, this above-optimized and validated UFLC-Qtrap-MS/MS method was applied to analyze the amounts of potential mycotoxins contained in 14 batches of Tartary buckwheat samples. As shown in Table 4, although all Tartary buckwheat seed samples were pre-sterilized by UV, regrettably, one of the 14 samples (Meigu No. 2) was found to be contaminated by AFB_1_, with a residual level higher than the regulatory MRLs suggested by EU. Figure 3 shows the MRM chromatogram of AFB_1_ residue in the contaminated Meigu No. 2 Tartary buckwheat sample, in which AFB_1_ occurred at (retention time) RT = 4.68 min. Therefore, the overproof result indicated that it was greatly necessary to determine the amounts of mycotoxins in Tartary buckwheat.

## 3. Conclusions

In view of the high rate of occurrence of fungal contamination in foodstuffs and the severe health threat of mycotoxins, it is critical to detect mycotoxin residues in Tartary buckwheat, considering its increasing application market. Thereofre, a simultaneous analysis method for multi-class mycotoxin determination with high sensitivity and selectivity, as well as lower time and cost requirements, was necessary. Herein, a simple and efficient UFLC-Qtrap-MS/MS method has been developed and validated for rapid identification and simultaneous quantification of 12 mycotoxins (AFB_1_, AFB_2_, AFG_1_, AFG_2_, HT-2, T-2, OTA, FB_1_, FB_2_, ZAN, ZON, and DON) in Tartary buckwheat. Among 14 batches of Tartary buckwheat cultivars collected from different origins, one moldy sample was found, with a high amount of AFB_1_ that exceeded the maximum residue limit in food suggested by the EU. The high carbohydrate content in Tartary buckwheat, along with unsuitable storage conditions, may result in fungal proliferation and mycotoxin residue. A simple and cost-effective approach for sample pretreatment without clean-up was established. This pretreatment procedure greatly improved the detection sensitivity, and was suitable for the complex Tartary buckwheat matrices. To the best of our knowledge, this was the first attempt to determine multiple mycotoxins in Tartary buckwheat. This quantitative analysis method provides a convenient approach for multi-class mycotoxin residue surveillance, with advantages including simple pretreatment, high selectivity and accuracy.

## 4. Materials and Methods

### 4.1. Chemicals and Reagents

Stock solution of aflatoxins containing 2 μg of AFB_1_, 2 μg of AFG_1_, 0.5 μg of AFB_2_, 0.5 μg of AFG_2_ in 1 mL of acetonitrile, together with powders (1 mg of each) of OTA, ZON, ZAN, FB_1_, FB_2_, HT-2, T-2 and DON toxins, were purchased from Pribolab (Biopolis, Singapore). The methanol and acetonitrile used as the mobile phase and extraction solvent were of HPLC grade, and were purchased from Honeywell (Burdick & Jackson, Sunrise Valley Drive, Reston, VA, USA). Other reagents and chemicals were of analytical grade, and were purchased from Beijing Chemical Works (Beijing, China). Water was double-distilled.

All glassware used was soaked in 5% aqueous sodium hypochlorite for several hours to destroy residual toxins before cleaning and reuse. After the analyses, all materials were decontaminated with 5% aqueous sodium hypochlorite solution.

### 4.2. Sample Collection and Preparation

14 batches of Buckwheat seeds produced in different districts (Cultivar Haizige buckwheat, Meigu No. 2, Da’anben No. 3, Liuku No. 3, Yunku No. 1, Jinku No. 2, Xiqiao No. 3, Jiujiang buckwheat, Yunnan huaku, Tongliao buckwheat, Chuanqiao No. 2, Heifeng No. 1, Dianning No. 1, Qianku No. 5 of *F. tataricum*) were obtained from the Key Laboratory of Coarse Cereals Processing, Ministry of Agriculture, Chengdu University (Chengdu, Sichuan, China). All seed samples were immediately transferred to sealed bags, to prevent humidity changes, and stored at 4 °C until analysis. One representative sample, Cultivar Xiqiao No. 3, from which the target compounds were absent, was used as a blank sample for method optimization and validation.

Tartary buckwheat seeds were pulverized into powder sufficiently fine to pass through a 50-mesh sieve. Finely ground sample was weighed (1 g) and placed in a 50 mL centrifuge tube. After addition of 10 mL methanol-water (8:2, *v*/*v*) the mixture was extracted by ultrasonic treatment. This extraction was conducted for 30 min. After centrifugation at 10,000 rpm for 5 min, 5.0 mL supernatant was immediately transferred to a 5.0 mL EP tube and evaporated under a stream of nitrogen gas at room temperature. Finally the residue was re-dissolved in 1.0 mL methanol-water (5:5, *v*/*v*) and the solution was filtered through a 0.22 μm filter for UFLC-QTrap-MS/MS analysis. The final concentration of the sample in the extract was 0.5 g·mL^−1^.

### 4.3. UFLC-QTrap-MS/MS Analysis of Multi-Class Mycotoxins

The Shimadzu ultra-fast liquid chromatography (UFLC) system (Shimadzu, Kyoto, Japan), equipped with two Shimadzu LC-20 AD pumps, a CBM-20A system controller, a SIL-20 AC auto-sampler and a CTO-20A column oven, was employed as the components separation role. The UFLC system coupled to a QTRAP^®^ 5500 mass spectrometer (AB SCIEX, Foster City, CA, USA) via a Turbo Ion Spray ionisation interface was used for the UFLC-QTrap-MS/MS analysis. Applied Biosystems Analyst software (version 1.6, AB SCIEX, Foster City, CA, USA) was used to control the UFLC-QTrap-MS/MS system and for data acquisition and processing. The UFLC separation was performed on a SHISEIDO Capcell core C_18_ column (2.1 mm × 50 mm, 2.7 μm) with a gradient elution with the flow rate of 0.35 μL·min^−1^ by a mobile phase consisting of 0.1% formic acid aqueous solution (A) and acetonitrile with 0.1% formic acid (B) with the following gradient: 0.00–1.50 min, linear change from 85% A to 80% A; 1.50–8.00 min, from 80% A to 5% A; 8.00–10.00 min, hold at 5% A; 10.00–10.01 min, switch from 5% A to 85% A and hold at 85% A for additional 3 min. The injection volume was 2.0 μL.

The MS worked with electrospray ionization (ESI) in positive and negative modes under the multiple reaction monitoring (MRM) condition. Curtain gas and source gas (GS 1 and GS 2) were set to 35, 55 and 55 psi, respectively. The spray voltage was 4500 V for positive ESI and −4500 V for negative ESI and the source temperature was set at 550 °C. In all cases, a precursor ion and two product ions (the most abundant for quantification and the other one for identification) were studied. Multiple reaction monitoring (MRM) mode was employed for quantitation.

### 4.4. Method Validation

The linearity, analytical limit, precision (intra- and inter-day variability), stability, recovery and matrix effect in this established method were evaluated to validate its reliability and accuracy.

Avoiding the matrix effect is highly critical for the determination of mycotoxins in foodstuffs, and plays an important role in the quantitative trace analysis. Specifically, bioactive molecules in Tartary buckwheat, such as polyphenols and flavonoids, could competitively interact with mycotoxins, leading to inaccuracy in the mass spectrometry signal. To investigate matrix effects, matrix-matched calibration curves were established by combining standard solutions into the blank Tartary buckwheat extracts to obtain a series of concentrations in the range of 1–200 μg·kg^−1^ for AFG_1_, AFB_1_ and OTA, 0.25–50 μg·kg^−1^ for AFG_2_ and AFB_2_, 5–1000 μg·kg^−1^ for FB_1_, FB_2_, HT-2, T-2, ZON and ZAN, 10–2000 μg·kg^−1^ for DON. In accordance with the previous method [26], the matrix effect was assessed by signal suppression/enhancement (SSE), which compared the peak area of mycotoxins in blank matrix with the peak area of mycotoxins in solvent. 

To evaluate the linearity of the calibration curves, the mixed standard solution was diluted with blank matrix into a series of concentrations, and was then injected into the UFLC/QTrap-MS/MS system. Relative standard deviation (RSD) values for each individual calibration level, calibration curve regression equations with their determination coefficients (r) were expressed, and linear range was determined. Quantification was carried out using external calibration method [27]. 

The sensitivity of the UFLC-QTrap-MS/MS analysis was evaluated using the limit of detection (LOD) and limit of quantification (LOQ). The LODs of the aflatoxins were estimated from blank samples spiked with decreasing concentrations of the analytes, where the response of the quantitative ion was equal to three times that of the blank samples. The LOQ was estimated in the same way as the LOD, but using the criterion of S/N ≥ 10 for the quantitative ion.

Intra- and inter-day variation was evaluated for the precision of the method. A blank Tartary buckwheat sample solution spiked with medium concentration (10 μg·kg^−1^ for AFG_1_, AFB_1_ and OTA, 2.5 μg·kg^−1^ for AFG_2_ and AFB_2_, 50 μg·kg^−1^ for FB_1_, FB_2_, HT-2, T-2, ZON and ZAN, 100 μg·kg^−1^ for DON) was analyzed by UFLC-Qtrap-MS/MS. The experiments were determined by six consecutive injections on the same day to calculate the intra-day precision, and by consecutive injection of the mixed samples at 0, 2, 4, 8, 16, 24, 48, 72 h to investigate the inter-day precision/stability, respectively.

The optimized extraction procedure was tested for recovery tests in blank samples at three (high, medium and low) spiked levels, which were 50, 10, 5 μg·kg^−1^ for AFG_1_, AFB_1_ and OTA, 12.5, 2.5, 1.25 μg·kg^−1^ for AFG_2_ and AFB_2_, 250, 50, 25 μg·kg^−1^ for FB_1_, FB_2_, HT-2, T-2, ZON and ZAN, 500, 100, 50 μg·kg^−1^ for DON. Then, the samples were extracted using the above-mentioned sample-preparation method, and each test was repeated in triplicate. The recovery (%) value in the blank matrix was calculated using the obtained calibration curves based on the following equation:

Recovery (%) = (measured concentration for spiked sample)/(spiked concentration) × 100

### 4.5. Statistical Analysis

All experiments were performed independently a minimum of three times, and data were expressed as mean ± standard deviation (SD). The statistical significance of the data was analyzed using Student’s *t*-test. 

## Figures and Tables

**Table 1:** UFLC-QTrap-MS/MS analysis parameters for 12 mycotoxins.

Analyte	RTW (min)	Precursor Ion (*m*/*z*)	Molecular Ion	Product Ions (*m*/*z*) ^a^	Product Ions Ratio ^b^ (mean ± RSD)	DP ^c^	EP ^c^	CE ^c^	CXP ^c^
AFG_2_	4.15–4.46	331.1	[M + H]^+^	245.1 (Q)	1.75 ± 0.03	130	10	40	17
				217.0 (q)		130	10	48	15
AFB_2_	4.38–4.69	315.0	[M + H]^+^	287.1 (Q)	1.32 ± 0.02	170	10	36	16
				259.1 (q)		170	10	40	16
AFG_1_	4.39–4.70	329.0	[M + H]^+^	243.1 (Q)	2.13 ± 0.05	130	10	37	14
				215.0 (q)		130	10	44	14
AFB_1_	4.60–4.90	313.0	[M + H]^+^	285.1 (Q)	2.09 ± 0.02	110	10	32	17
				269.0 (q)		110	10	43	17
FB_1_	4.26–4.56	722.5	[M + H]^+^	334.5 (Q)	1.06 ± 0.03	120	10	52	14
				352.4 (q)		120	10	48	13
FB_2_	4.71–5.02	706.5	[M + H]^+^	336.2 (Q)	3.51 ± 0.02	110	10	47	15
				318.5 (q)		110	10	43	15
HT-2	4.78–5.09	425.1	[M + H]^+^	263.0 (Q)	1.48 ± 0.15	90	10	15	13
				215.0 (q)		90	10	15	10
T-2	5.60–5.85	467.2	[M + H]^+^	215.0 (Q)	1.16 ± 0.12	110	10	20	11
				185.1 (q)		110	10	24	13
OTA	5.91–6.18	404.2	[M + H]^+^	239.1 (Q)	3.93 ± 0.05	110	10	30	13
				358.0 (q)		110	10	26	13
DON	1.09–1.60	341.0	[M − H]^−^	265.2 (Q)	1.15 ± 0.03	−70	10	−16	−11
				295.2 (q)		−70	10	−15	−12
ZON	5.92–6.28	317.1	[M − H]^−^	131.0 (Q)	1.94 ± 0.02	−70	10	−41	−11
				175.0 (q)		−70	10	−25	−7
ZAN	5.87–6.15	319.1	[M − H]^−^	205.0 (Q)	5.18 ± 0.14	−180	10	−34	−15
				275.0 (q)		−180	10	−40	−15

^a^ Product ions: Q, quantifier transition ion; q, qualifier transition ion. ^b^ Peak-area ratio of quantifier and qualifier transition ions in the matrix Cultivar Xiqiao No. 3. ^c^ DP, declustering potential; EP, entrance potential; CE, collision energy; CXP, collision cell exit potential. All expressed in volts.

**Table 2:** Linearity, LOD, LOQ and SSE for the test of 12 mycotoxins by UFLC-QTrap-MS/MS determination.

Mycotoxin	Linear Equation	r	Linear Range (ng·mL^−1^)	LOQ (ng·mL^−1^)	LOD (ng·mL^−1^)	SSE (%)
AFG_2_	Y = 1.58 × 10^4^X − 405	0.9993	0.5–25	0.5	0.25	119.21
AFB_2_	Y = 3.85 × 10^4^X − 182	0.9997	0.25–25	0.25	0.125	95.20
AFG_1_	Y = 4.58 × 10^4^X − 9.55 × 10^3^	0.9992	1–100	1	0.5	114.85
AFB_1_	Y = 6.05 × 10^4^X + 3.06 × 10^4^	0.9993	1–100	0.1	0.05	111.82
FB_1_	Y = 2.2 × 10^3^X − 1.4 × 10^3^	0.9988	5–500	5	2.5	102.47
FB_2_	Y = 4.35 × 10^3^X − 729	0.9989	5–500	1	0.5	113.20
HT-2	Y = 201X + 512	0.9982	50–500	50	20	103.68
T-2	Y = 278X + 339	0.9974	50–500	50	20	112.40
OTA	Y = 3.45 × 10^4^X − 1.78 × 10^3^	0.9998	1–100	0.25	0.1	116.88
DON	Y = 358X + 1.2 × 10^3^	0.9999	50–1000	50	25	121.80
ZON	Y = 1.06 × 10^4^X − 1.25 × 10^4^	0.9994	5–500	1	0.5	112.09
ZAN	Y = 1.68 × 10^3^X − 2.6 × 10^3^	0.9999	10–500	10	5	113.22

**Table 3:** Recoveries (*n* = 3) of the UFLC-Qtrap-MS/MS method for mycotoxins in matrix of Tartary buckwheat.

Mycotoxins	High Level (50 μg·kg^−1^)		Medium Level (10 μg·kg^−1^)		Low Level (5 μg·kg^−1^)	
	Mean (%)	RSD (%)	Mean (%)	RSD (%)	Mean (%)	RSD (%)
AFG_2_	79.77	11.25	80.20	10.49	79.52	13.85
AFB_2_	82.52	9.66	87.66	12.62	83.05	10.71
AFG_1_	88.58	6.94	90.48	7.40	95.20	5.29
AFB_1_	100.79	5.62	103.25	6.91	104.24	4.80
FB_1_	102.45	11.28	99.68	14.05	105.24	13.92
FB_2_	92.82	10.29	86.86	12.54	96.16	12.92
HT-2	103.60	13.77	105.27	12.58	108.92	12.94
T-2	96.97	10.61	89.81	9.42	94.70	11.38
OTA	95.39	4.35	98.50	5.77	106.85	5.28
DON	103.27	9.56	106.75	13.52	108.39	14.27
ZON	96.71	4.78	93.28	6.93	94.61	8.54
ZAN	94.62	4.96	87.05	8,55	90.52	7.62

**Table 4:** Occurrence and residual level of mycotoxins in 14 batches of Tartary buckwheat samples from different districts.

Sample	Origin	Mycotoxin Detected	Mycotoxin Residue (μg·kg^−1^)	MRL Suggested (μg·kg^−1^)
Cultivar Haizige	Yanyuan city, Sichuan Province	ND ^a^	-	
Meigu No. 2	Meigu city, Sichuan Province	AFB_1_	5.62	˂2.0 μg·kg^−1^ for AFB_1_˂4.0 μg·kg^−1^ for sum of Afs ^b^
Da’anben No. 3	Meigu city, Sichuan Province	ND	-	
Liuku No. 3	Jintang city, Sichuan Province	ND	-	
Yunku No. 1	Jintang city, Sichuan Province	ND	-	
Jinku No. 2	Jintang city, Sichuan Province	ND	-	
Xiqiao No. 3	Jintang city, Sichuan Province	ND	-	
Jiujiang buckwheat	Dayi city, Sichuan Province	ND	-	
Yunnan huaku	Meigu city, Sichuan Province	ND	-	
Tongliao buckwheat	Yanyuan city, Sichuan Province	ND	-	
Chuanqiao No. 2	Jintang city, Sichuan Province	ND	-	
Heifeng No. 1	Dayi city, Sichuan Province	ND	-	
Dianning No. 1	Yanyuan city, Sichuan Province	ND	-	
Qianku No. 5 of *F. tataricum*	Dayi city, Sichuan Province	ND	-	

^a^ Not detected. ^b^ Sum of AFB_2_, AFB_1_, AFG_2_ and AFG_1_.
